# Chronic Invasive Fungal Granuloma–A Diagnostic Dilemma in an Immunocompetent Host

**Published:** 2016-01

**Authors:** Shrinivas S. Chavan, K.S. Bhople, Sunil D Deshmukh, Prateek V. Jain, Mangala Sonavani

**Affiliations:** 1 *Department of Otorhinolaryngology, Government Medical College, Aurangabad, India.*; 2 *Department of Pathology, Government Medical College, Aurangabad. Maharashtra State, India.*

**Keywords:** Chronic invasive granulomatous fungal sinusitis, Immunocompetent Host, Tuberculoma and Chronic Granulomatous disease

## Abstract

**Introduction:**

Invasive fungal sinusitis, though considered to be rare entity, is nowadays frequently encountered, not only in immunocompromised patients but also in immunocompetent patients. The changing prevalence towards immunocompetent hosts is due to the indiscriminate usage of broad spectrum antibiotics, steroids, and immunosuppressive drugs. Diagnosing invasive fungal sinusitis should not pose any difficulty to both the clinician [a whitish colour secretion in elderly Diabetics, and CT Scan PNS showing concretion in the sinus along with destruction of the surrounding bone] and to the pathologist; however, when the invasive fungal sinus infection presents in a form of a granuloma then its diagnosis imposes a challenge to medical professionals.

**Case Report:**

We are presenting a case study,which consists of 3 cases of chronic invasive fungal sinus infection.Two patients were treated for tuberculoma and had completed a course of Anti Koch’s Treatment and one patient was given a trial of broad spectrum antibiotics and steroids.Eventually all cases were diagnosed as a chronic invasive form of fungal granuloma (CIFG).

**Conclusion:**

CIFG of the paranasal sinuses is seen in immunocompetent hosts, especially those that are in the 2nd and 3rd decades of their lives. Gradually progressive proptosis is the primary presenting symptom. MRI scanning is a better imaging modality compared to CT scanning. Routine H&E staining may prove inadequate and special stains such as the GMS stain should be employed in the slightest doubt of a fungal aetiology. A team approach towards patients is paramount for early diagnosis and timely medical and surgical intervention.

## Introduction

Fungi are omnipresent eukaryotic organisms mostly transmitted by air, which, in certain conditions can impose morbidity in the upper air way. It is the interaction between the index of pathogenecity of fungal species, immunological status of the host, and overall complex cellular and humoral response favoring the fungus to gain a foothold in the nasal and sinus cavities that causes fungal rhinosinusitis. Fungal organisms present in air are often inhaled, but the effective functioning of the immune system normally kills these. Invasive fungal infectionsare seen both in immunosuppressed and immunocompetent hosts, but CIFG usually presents in immunocompetent patients. Granuloma formation is causedby an inherited disorder of the immune system, affecting a specific part of the immune system,which results in the inability to produce the group of chemicals (Reactive oxygen species) used by the immune system to kill invading microorganisms such as Aspergillus (1).The Granuloma is the last resort response, where the immune system tries to localize and prevent an infective or irritant stimulus by walling it off with a compact aggregate of histiocytes. As a result microorganisms tend to persist and form a granuloma as the other cells of the immune system accumulate around the microorganism, preventing its spread in the body. CIFG rarely occurs in the world, but it does show predilection towards hot dry climatic countries like Sudan, India, Pakistan and Saudi Arabia (2).

In developing countries, especially in tertiary centres, pathologists primarily use the Haematoxylin & Eosin (H&E) stain for granulomatous lesions. On many occasions, crowding of epitheloid cell, Langhans giant cell (LGC), plasma cells with and without caseous necrosis overshadows the sparsely distributed fungal element resulting in erroneous reporting of chronic granulomatous lesion and the tendency of not investigating further is justified by the high prevalence of TB in developing countries. Therefore, the patient is often subjected to AKT due to histopathological presumptions.

Initially, patients show a dramatic improvement with antibiotics. The probable explanation is the subsidence of inflammatory response to fungal infection. As the granuloma subsides, which is otherwise helping the host to limit the spread of fungal infection by entrapping it, the fungus spreads more aggressively and the initial improvement shown by the patient is deteriorated in due course. Due to our knowledge about the changing spectrum of CIFG, this present study is a sincere attempt to chronicle the necessity for the otorhinolaryngologist, radiologist and pathologist to work as a team in cases of invasive chronic granulomatous diseases of the paranasal sinus (PNS).

***Case Report 1***

**Fig 1:**
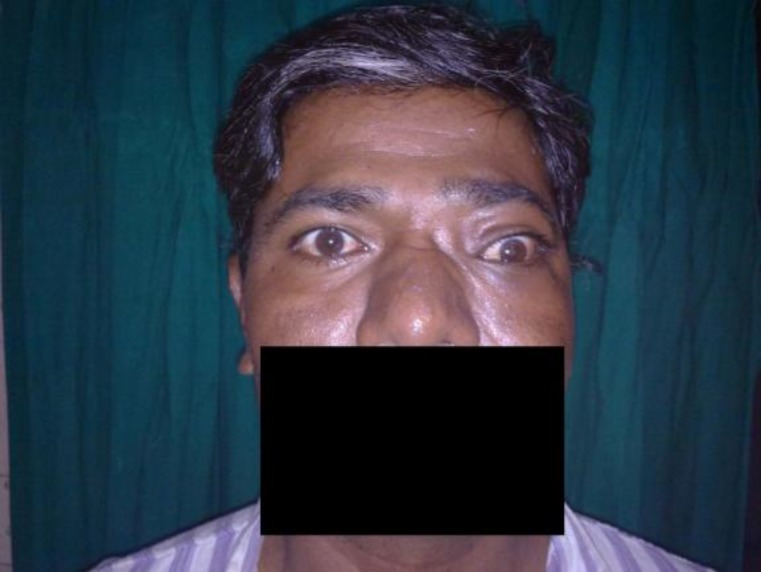
Photograph at presentation (Case 1

A 28-year-old male was presented to the otorhinolaryngology department, with 6-month history of left sided proptosis with restricted extra ocular movement (Fig.1).

**Fig 2:**
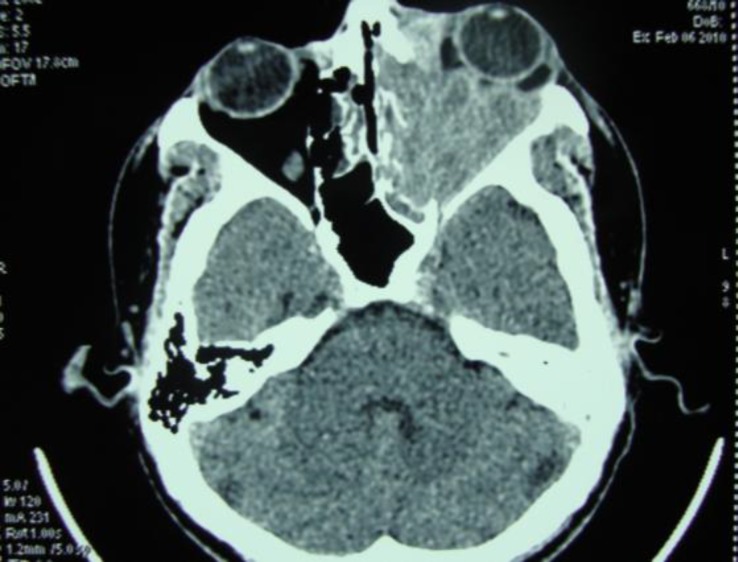
CT Scan PNS showing maxillary mass and proptosis (Case 1

There was no h/o of vision loss and trauma. The nasal examination showed a whitish secretion in the nasal meatus. Haemogram showed raised ESR. The CT Scan PNS showed a mass occupying the maxillary sinus and eroding the infraorbital plate and it was reported as a neoplasm (Fig. 2).

**Fig 3:**
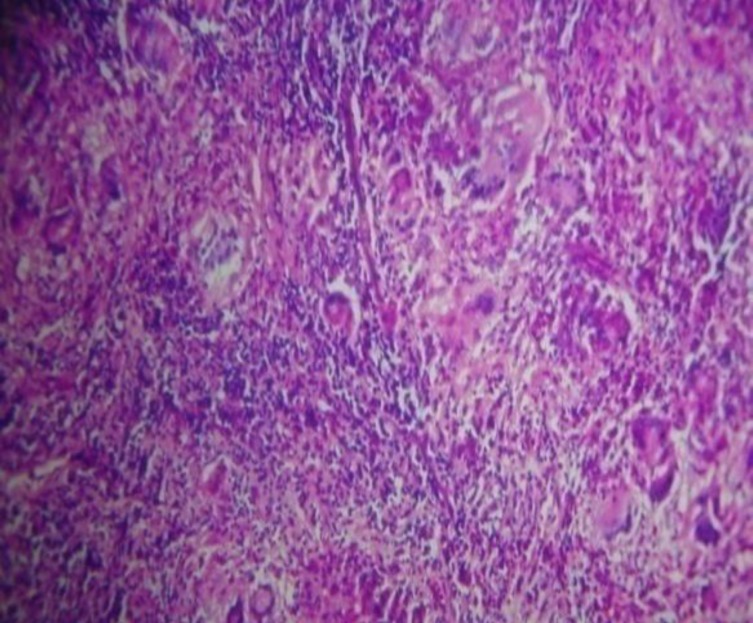
Histopathologial microphotograph showing Langhans Giant Cells, Epitheloid Cells suggestive of Tuberculoma (Case 1

The patient underwent a FESS procedure and the mass was removed in bits and pieces with minimal intraopertive bleed. The mass was sent for Histopathology and was reported as a Tuberculoma (Fig.3).

**Fig 4:**
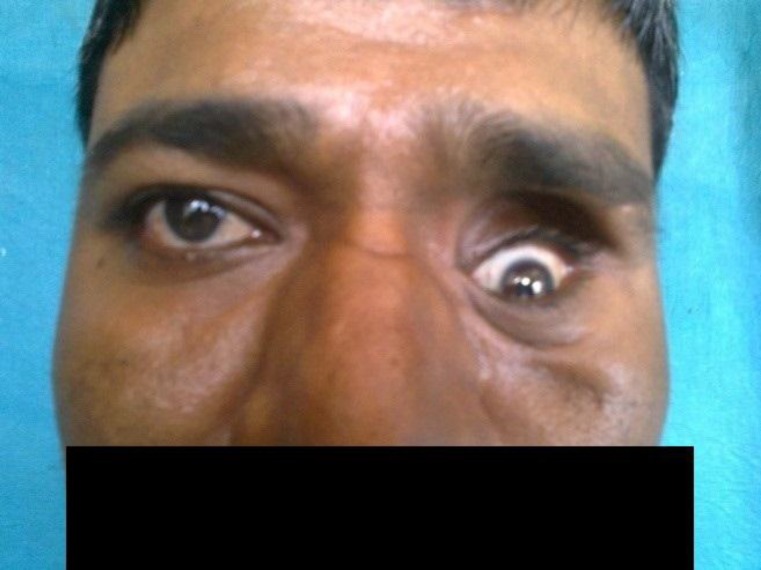
Photograph after antifungal treatment (Case 1

The patient was started on AKT with regular follow up. The patient completed a 9-month course of AKT and was symptom free in his follow up but later developed similar complaints of left sided proptosis. The patient was again posted for endoscopic biopsy. The pathologist used the Gomori’s methanamine silver nitrate (GMS) stain and reported it as an invasive granuloma of Aspergillus fungus. The patient was started on an antifungal drug with regular follow up and finally improved (Fig. 4).

***Case Report 2***

**Fig 5:**
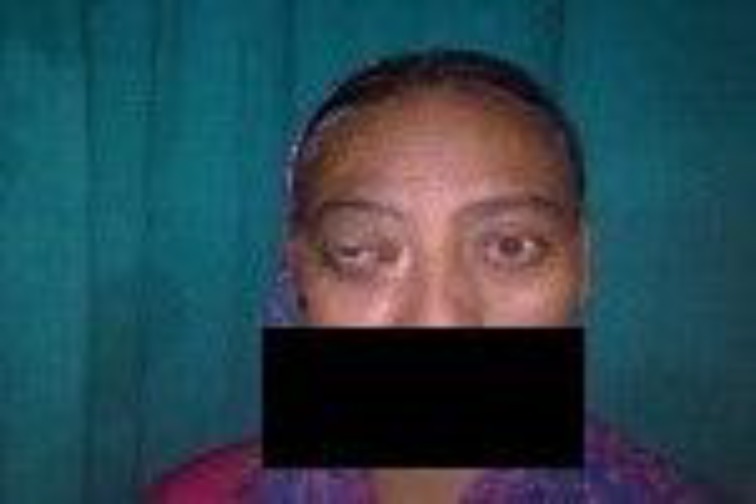
Photograph at presentation (Case 2

**Fig 6:**
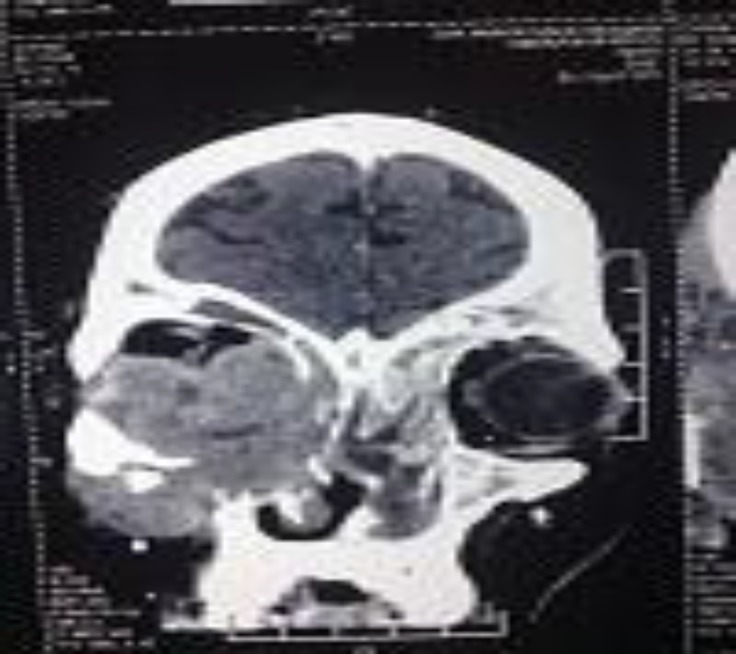
CT Scan PNS Coronal View showing intraocular involvement (Case 2

A 32-year-old female came with the H/O of right sided proptosis and restricted extraocular movements (Figs.5,6).

**Fig 7:**
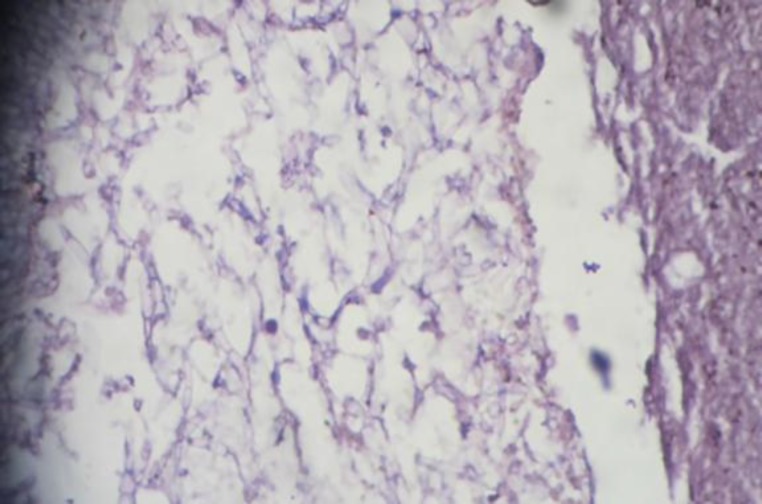
Microphotograph showing Aspergillus (Case 2

The patient was a diagnosed case of Tuberculoma and was started on AKT in a private institute. She had come to our institute to register her dose for AKT. Her symptoms worsened during her follow up in our department. She was posted for FESS and the mass was sent for HPR. The HPR picture showed an increase in epitheloid cells, LGC, and caseous necrosis on H&E stain. Considering our previous experience, on this occasion GMS stain was used to identify distributed fungi in a necrotizing granuloma (Fig.7).

**Fig 8:**
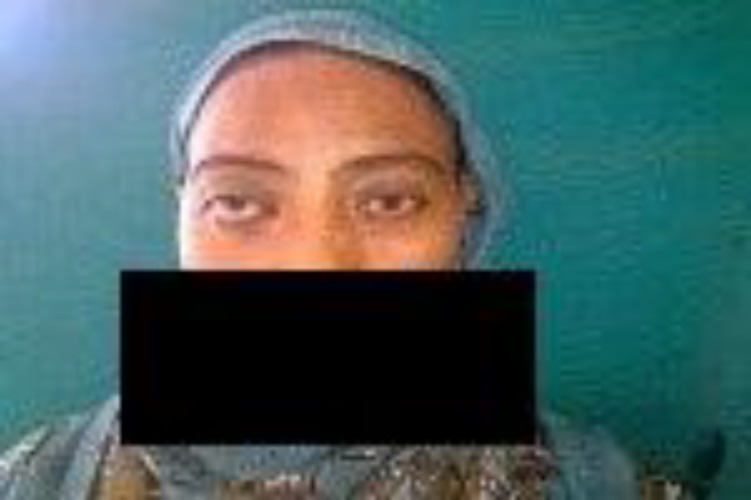
Photograph after antifungal treatment(Case 2)

The case was diagnosed as CIFG and she was started on Amphotericin B and isnow doing well (Fig.8).

***Case Report 3***

**Fig. 9:**
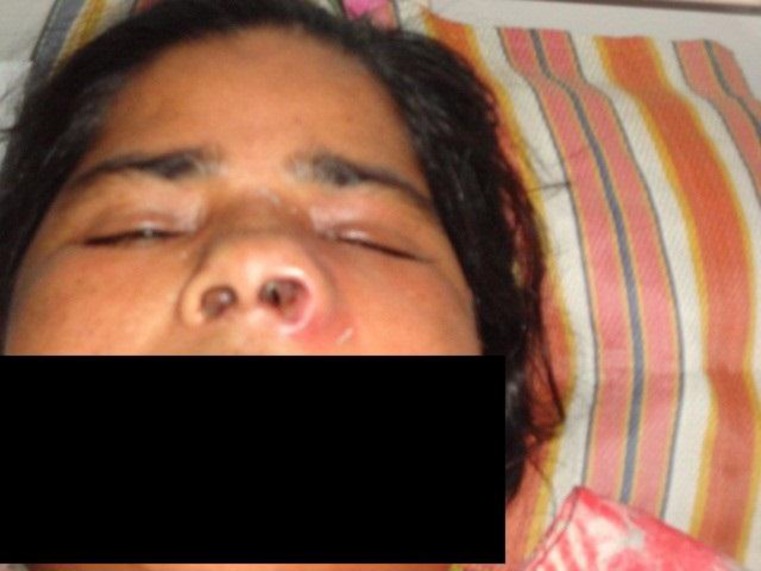
Photograph at presentation (Case 3

**Fig10:**
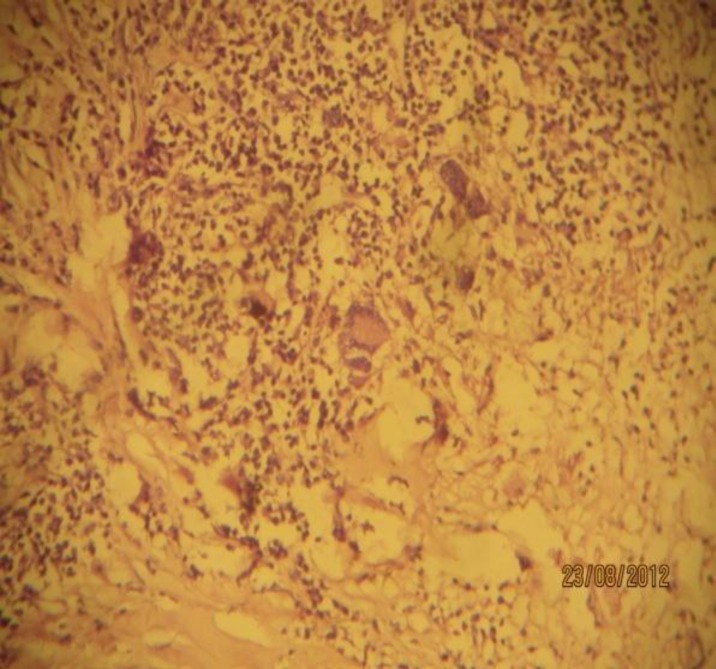
HPR showing Epitheloid cell & LGC without necrosis (Case 3

A 28-year-old female came with H/O of left sided slow growing cheek swelling (Fig.9). The CT Scan PNS showed a mass occupying the maxillary sinus and ethmoid sinus with septal erosion and invasion into the opposite nostril. Haemogram showed raised ESR and her ANCA were negative. The patient underwent a biopsy and the tissue was sent for HPR. The HPR showed epitheloid cell & LGC without necrosis (Fig.10).

**Fig 11:**
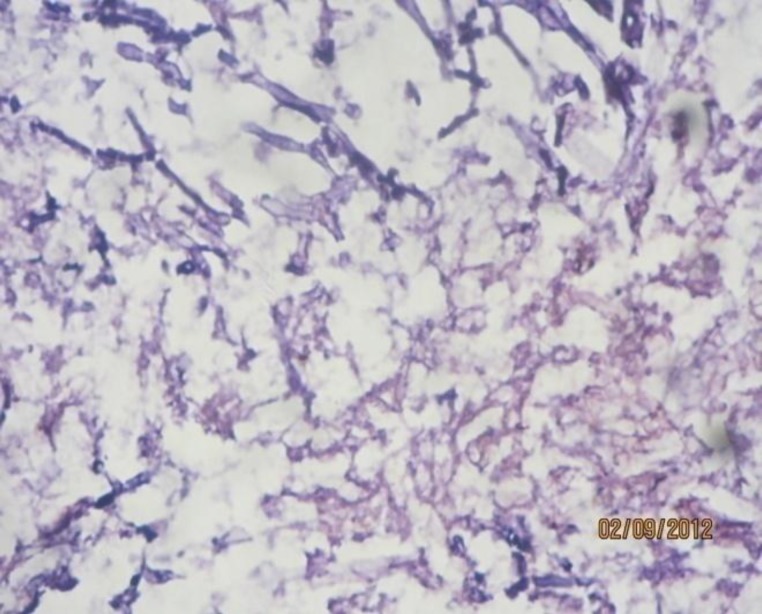
HPR showing Aspergllosis

The patient was treated for a chronic granulomatous disease of the nose and given a trial of broad-spectrum antibiotics and steroids. The patient was referred to a higher center as there was no significant improvement. She was diagnosed as an invasive form of Aspergillus granuloma (Fig. 11). Unfortunately, the patient died of systemic illness.

## Discussion

Cases of Fungal Rhinosinusitis are on the rise and are being reported from all corners of the world. Patients show a wide array of symptoms, with a complex tug-o-war between the fungal virulence and host defense mechanisms, ranging from allergic fungal sinusitis to invasive and noninvasive fungal sinusitis. Allergic fungal sinusitis is characterized by Charcot Leyden crystal and raised IgE level (3).

Invasive fungal sinusitis is mostly seen in immunosuppressed patients, but in this case report, all patients were immunocompetent and invasive fungal sinusitis presented itself in a form of granuloma. Two cases presented with proptosis as their main complaint and one case presented with slowly growing cheek swelling. These findings coincide with the study done by N.K. Panda et al in 1996 Raised ESR was a nonspecific finding (4).

The CT Scan PNS was done in all cases, which showed erosion of surrounding structures and accordingly, were reported as a malignancy of the PNS. The radiological features of CIFG correspond with three separate studies conducted in past (4-6).

On HPR, all the cases showed the characteristic feature of increase epitheloid cell & LGC. In addition, necrosis was seen in two cases. This is in accordance with the HPR of a case study done by Sanjay Mukhopadhyay, where fungi was sparsely present amidst the necrotic granulomatous lesion, and therefore were missed and reported as a tuberculoma(7). Similar citations were observed by G. Surya Prakash Rao et al in 1982 (8).

The two patients diagnosed with tuberculoma were started on AKT. One patient completed a 9-month course of AKT and was doing well in his follow up; however, his symptoms recurred on subsequent follow up and the patient underwent a repeat endoscopic biopsy. The GMS stain was used, which lead to the detection of aspergillus, paving way to the diagnosis of CIFG.

The other patient was started on AKT and showed symptomatic improvement for the first 2 months of treatment; but later showed deterioration in her condition. She underwent a biopsy and on GMS stain, showed aspergillus, ending the diagnostic quest.

The final case was treated for a chronic granulomatous lesion and was given a trial of broad-spectrum antibiotics and steroids. However, similarly to prior cases, her symptoms flared up after initial improvement and she was referred to a higher center where she was diagnosed with invasive aspergillosis. 

All the cases showed Aspergillus species in Invasive granuloma, which coincides with the study conducted by N. K. Panda et al (4).

This case study is a sincere attempt to create awareness in clinicians about the emerging trends of clinical presentations and among radiologist to go for an MRI scan, which shows high intensity signal on T1 weighted image for malignancy and low intensity signal for CIFG and among pathologists to consider the use of special stains while examining any necrotizing granulomatous tissue of the PNS and collectively work as a team to prevent delaying the diagnosis of CIFG and save time by starting patients on an antifungal regime as early as possible (9,10).

## Conclusion

1. CIFG of the paranasal sinuses is seen in immunocompetent hosts, especially those in the 2^nd^ and 3^rd^ decade of their lives.

2. Gradually progressive proptosis is the primary presenting symptom.

3. MRI scanning is a better imaging modality compared to CT scanning.

4. Routine H&E staining may prove inadequate and special stains such as the GMS stain should be employed in the slightest doubt of a fungal aetiology. 
